# Keratitis caused by *Nocardia farcinica* in a contact lens wearer

**DOI:** 10.1016/j.imj.2022.04.001

**Published:** 2022-04-28

**Authors:** Shaymaa Hegazy, Tung Phan

**Affiliations:** Department of Pathology, University of Pittsburgh, Pittsburgh, PA, USA

**Keywords:** Keratitis, *Nocardia farcinica*, Contact lenses, Sequencing

## Abstract

Bacterial keratitis is an infection of the cornea. This kind of infection can progress quickly and if left untreated, it can eventually cause complete blindness. While *Staphylococcus aureus* and *Pseudomonas aeruginosa* are most commonly responsible for this type of infection in the United States, *Nocardia* spp. is rarely encountered. Here we describe an interesting case of *Nocardia farcinica* keratitis in a 31-year-old male patient having an extended wear of contact lenses. The patient presented at the emergency department with irritation, foreign body sensation, redness, and pain of his left eye. *Nocardia farcinica* was isolated from a corneal scraping specimen, and determined by sequencing a region of 16S rRNA gene. The patient had antimicrobial therapy with good improvement. We highlight the important role of *Nocardia farcinica* in causing keratitis, and its accurate and timely diagnosis is needed to avoid poor visual outcomes.

## 1 Introduction

Members of the genus *Nocardia* are aerobic actinomycetes that are ubiquitous in environments such as decaying organic matter, soil, fresh, and salt water [1,2]. People may become infected with *Nocardia* when they inhale it or when it enters an open wound, and then possibly spreads throughout the body known as a disseminated infection [3,4]. There are at least 100 different *Nocardia* species (https://lpsn.dsmz.de/genus/nocardia), and more than half of which have been shown to be medically significant [5]. Among them, *Nocardia asteroides* is the most common human pathogen in the United States where approximately 500–1000 cases of nocardiosis per year were recorded in the early 1970s, but the actual number is expected to be higher as of today [6,7]. Two common sites of *Nocardia* infection (nocardiosis) are lungs and skin, but it frequently disseminates to other locations including the central nervous system [8,9]. A small number of human cases of arthritis, septicemia, granulomatous hepatitis, paravertebral abscess, and pericarditis caused by *Nocardia* have been reported [8], [9], [10], [11], [12], [13], [14]. Compared to other species, *Nocardia farcinica* has a higher risk of dissemination to the brain [15]. The prognosis is often poor, and the mortality rate is high, up to 31% [16]. Gnanam et al. reported 43 patients diagnosed with *Nocardia* endophthalmitis in 10 years in India. Of note, *Nocardia farcinica* was the most predominant species (45%) causing endophthalmitis, followed by *Nocardia cyriacigeorgica* (18%) [17].

## 2 Case presentation

**Fig. 1:**
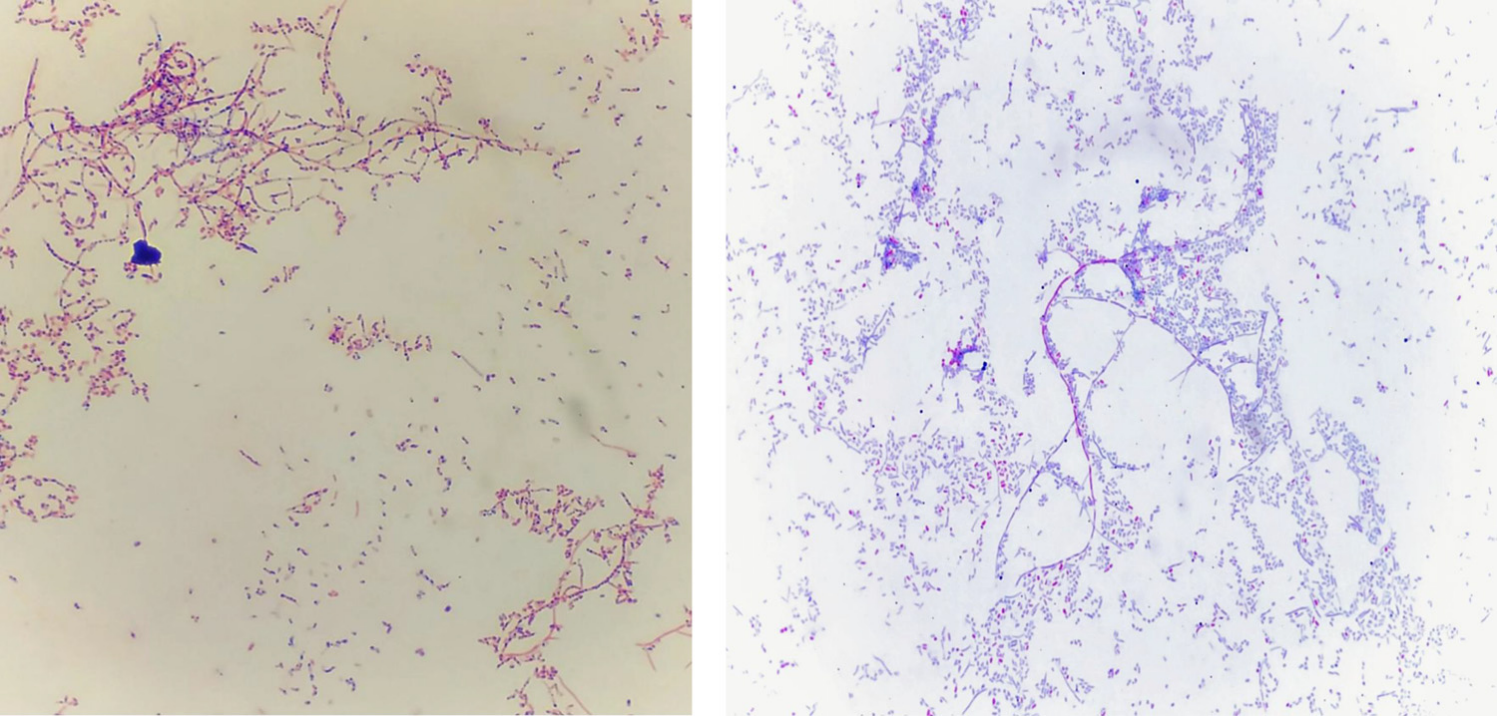
The photograph is of *Nocardia farcinica* only. Microscopic examination of a Gram-stained smear (left) and a modified acid-fast smear (right) of *Nocardia farcinica* that revealed branching, filamentous and beaded Gram-positive bacilli at 1000 × magnification.

A 31-year-old male patient with a past medical history of gastroesophageal reflux and plantar fasciitis. The patient is a firefighter contact lens wearer who presented at the emergency department with irritation, foreign body sensation, redness, and pain of his left eye. The patient reported that he was swimming and wearing his contact lenses in the hot tub and pool around 1 week earlier of his presentation. In addition, he typically wore his contact lenses for 10 days straight without removing them for sleep. He was diagnosed with a corneal abrasion and discharged on ciprofloxacin eye drops every 2 hours. The patient was also referred to an optometrist on the following day. The eye examination with slit lamp biomicroscopy demonstrated 3 corneal ulcers including a 0.25 mm × 0.25 mm central ulcer with epithelial ridge defect and 2 paracentral infiltrates measuring 0.5 mm × 0.5 mm and 0.75 mm × 0.75 mm. The remainder of the eye examination was unremarkable. Based on the atypical clinical appearance of the patient's ulcers, ciprofloxacin eye drops were discontinued. The patient was started on moxifloxacin eye drops every 2 hours and erythromycin ointment at night. However, his symptoms continued to persist, with progressively worsening blurriness, photosensitivity, redness and pain of his left eye. A couple of days later, the patient was seen by an ophthalmologist. His eye examination showed the same lesions. The patient was prescribed on tobramycin and cefazolin eye drops every 1 hour. A corneal scraping specimen was also collected and submitted to our clinical ophthalmic microbiology laboratory for bacterial and fungal cultures. After 48 of incubation at 35 °C in 5% CO_2_, growth was observed on nonselective blood and chocolate agar plates. The aerobic culture grew chalky white colonies that, when Gram stained, showed filamentous beading and branching Gram-positive organism (Fig. 1). A Ziehl-Neelsen modified acid-fast stain demonstrated that the organism was weakly acid fast positive, suspicious for *Nocardia* (Fig. 1). The further identity of *Nocardia farcinica* was determined by sequencing a region of 16S rRNA gene at Mycobacteria/Nocardia Laboratory, University of Texas Health Science Center at Tyler. Subsequently, the tobramycin eye drops were discontinued, and the cefazolin eye drops was decreased in frequency into every 4 hours. One week later, at the time of follow-up, the patient clinically improved with better vision.

## 3 Discussion

Though *Nocardia* is found worldwide, keratitis caused by *Nocardia* is rare. *Nocardia* keratitis is characterized by slowly progressive clinical course and poor visual outcomes. The diagnosis of *Nocardia* keratitis is often missed or delayed due to the rarity [2,6]. In addition, the clinical presentation of keratitis is often similar between *Nocardia* and other pathogens such as fungi, viruses and parasites. *Nocardia* keratitis is sometimes resistant to typical first-line ocular antibiotics. Prompt diagnosis is important to avoid suboptimal outcomes such as blindness. Most of the time, keratitis comes from ocular exposure to soil or plant matter or from corneal trauma [18]. Other known risk factors are ocular surface diseases, prior ocular surgeries (including corneal transplants), and topical steroid use [8]. Herein, we presented a case of *Nocardia farcinica* keratitis in a 31-year-old male patient who had an extended wear of contact lenses without a history of corneal trauma or any other risk factors. The patient had a past medical history of gastroesophageal reflux and plantar fasciitis, which did not play any role in the development of *Nocardia farcinica* keratitis. Extended contact lens wear is a major risk factor of infectious keratitis, due to its interference with the natural defense mechanisms of the ocular surface [19]. Lam et al. reported 223 patients having keratitis over a period of 17 months in Hong Kong [20]. It was found that wear of contact lenses was responsible for 26% of cases. While *Pseudomonas aeruginosa* was the most important pathogen (31%), only a single case of *Nocardia* was identified [20]. Even though extremely rare, *Nocardia farcinica* keratitis has also been described in children. Verner et al. reported the first case of *Nocardia farcinica* in a pediatric patient who used contact lenses while swimming in a pool [21]. *Nocardia* is considered as an opportunistic pathogen, which causes many infections in immunosuppressed patients. However, approximately one-third of infected patients are immunocompetent as also seen in our patient. Accurate diagnosis and prompt treatment are critical for *Nocardia* keratitis and ultimately lead to better patient outcomes.

## Author contributions

TP and SH: designed the study and wrote the manuscript.

## Declaration of competing interest

The authors declare no competing financial interests.

## Funding sources

This research did not receive any specific grant from funding agencies in the public, commercial, or not-for-profit sectors.
